# Impacts of User Personality Traits on Their Contributions in Idea Implementation: A Moderated Mediation Model

**DOI:** 10.3390/bs14030210

**Published:** 2024-03-06

**Authors:** Xuejiao Mi, Huiying Zhang, Fei Qu

**Affiliations:** 1 College of Management & Economics, Tianjin University, Tianjin 300072, China; tjumxj2023@163.com (X.M.); hyzhang@tju.edu.cn (H.Z.); 2 School of Management, Guilin University of Technology, Guilin 541004, China

**Keywords:** personality traits, user contributions in idea implementation, user engagement, China

## Abstract

In the realm of open innovation, users have emerged as a significant external source of innovation that enterprises cannot afford to overlook. Implemented ideas play a pivotal role in driving the iterative innovation of products within enterprises. However, the existing literature still lacks an exploration of specific impact mechanisms on contributions in idea implementation. This study presents a model that delineates the impact of user personality traits on idea implementation contributions, drawing upon theories such as personality trait theory, user engagement perspective, and trait activation theory. Empirical research was carried out by utilizing user data obtained from the Chinese high-tech company Xiaomi’s MIUI community. Personality trait indicators were developed through the application of text mining and machine learning techniques. To evaluate the models, a negative binomial regression model, which is well-suited for handling discrete data, was employed. The findings of this study indicate that user openness and conscientiousness positively influence their idea implementation contribution, whereas neuroticism has a negative impact on implementation contribution. Additionally, it is observed that user engagement plays a partial mediating role in the relationship between openness, conscientiousness, neuroticism, and idea implementation contribution. Community incentives can positively moderate the impact of user engagement on the relationship between conscientious personality and idea implementation contribution. This study expands the analysis of the impact mechanism of user idea implementation contributions, which has important theoretical guidance and practical implications for accurately identifying leading users in open innovation communities and enhancing user innovation contributions.

## 1. Introduction

With the rapidly changing and competitive market environment in the Internet era, enterprises need to identify, connect, and use external knowledge sources as the core innovation process to improve enterprise performance. Users with different skills and professional backgrounds can provide enterprises with leading innovative ideas to improve their products and services [1,2]. More and more enterprises have increased their investment in the open innovation community, trying to crowdsource new ideas from users [3,4]. Prominent examples include BMW’s Co-Creation Lab platform, LEGO’s Ideas platform, and Chinese Xiaomi’s MIUI new product development community. However, some studies indicate that enterprise decision-makers seriously underestimate the value of users as a source of innovation [5], and the significance of users’ contributions to innovation warrants consideration.

The information on user innovation in the open innovation community is mixed [6]. Prior research has primarily concentrated on considering the number of ideas proposed by users as innovative contributions, neglecting to systematically evaluate the quality of user creativity. In fact, only ideas that are embraced by enterprises can drive iterative innovation and hold genuine value. Consequently, we define user innovation as the contribution of users in idea implementation [6,7,8,9,10]. There remains a dearth of studies that acknowledge the significance of user personality traits among the factors that affect user contributions [11,12]. A personality trait is a psychological structure that can make people’s behavior tendency show the characteristics of persistence, stability, and consistency, which reflects the consistency and regularity of individual behavior [13]. The open innovation community is an internet-based virtual network platform created by enterprises [14]. It diverges from the structure of traditional enterprise organizations, as it is distinguished by the voluntary engagement of users, absence of central authority, and an atmosphere of freedom [15]. These distinctive features foster an environment that facilitates the expression of individual personality traits. Taking the representative Xiaomi community as an example, according to the 2021 financial report, the global monthly active users of MIUI have surpassed 500 million, indicating a significant source of innovation resources. Undoubtedly, the innovation contributions made by individuals possessing diverse personality traits within the MIUI community exhibit significant variance. Consequently, it becomes imperative to explore the extent and manner in which varied personality traits influence their contributions in idea implementation within open innovation communities.

This study aims to address the following three issues. Firstly, personality traits have been widely used in sociology and management to explain individual life and work behaviors [16,17,18,19,20], this study seeks to explore the impact of the various personality traits exhibited by users in open innovation communities on their levels of contribution. Furthermore, an increasing amount of academic research indicates that individual personality traits may indirectly affect such outcomes through various intermediary variables [21,22]. In exploring the specific influencing mechanisms, the notion of “user engagement” has gained recognition. This notion is derived from the marketing concept of “customer engagement”. Through interactive experiences, users establish ongoing emotional connections with enterprises or platforms by sharing comments and exchanging experiences, thus becoming co-creators of value by contributing their insights [23]. And it becomes evident that users who strongly align with an enterprise are more likely to recognize its value and generate more innovative contributions in community interactions. Introducing this concept aids in comprehending the influence of user personality traits on contributions in idea implementation. Thirdly, this study seeks to investigate the moderating influence of community incentives on the association between personality traits and user engagement, thereby elucidating the impact of external contexts on the manifestation of personality traits. In essence, the objective is to enhance and expand existing research on the conditions under which personality traits influence contributions in idea implementation by employing moderated mediation models.

This study makes three significant contributions to the field. (1) It offers an interdisciplinary framework for examining the influence of personality traits on user contributions in idea implementations. These findings expand the interactive application of psychology and innovation management field [24]. (2) It introduces a user engagement perspective. It introduced the perspective of user engagement, improved the research on the impact mechanism from user personality traits to contributions in idea implementation, and supplemented the gap in the previous research on the lack of individual-level impact mechanisms. By exploring the moderating effect of community incentives on personality traits and user engagement, this study contributes to a deeper comprehension of the contextual factors at play. (3) Traditional research employs the questionnaire survey method to identify personality traits, which has been called into question due to its low response rate and difficulty in ensuring objective authenticity. The growing integration of social science and computer science has prompted researchers to develop automated methods to predict personality traits [25,26], This study proposes an indicator method to quantify personality traits through users’ text messages, which has a certain exploration for the combination of econometrics and machine learning and also provides an objective reference basis different from previous researchers for personality trait quantification. This research not only aids the theoretical explanation of user innovation behavior under the influence of different personality traits of users but also provides practical guidance for the open innovation community to better organize leading users and build community activities.

## 2. Theoretical Foundation and Research Hypotheses

### 2.1. User Contributions in Idea Implementation within the Open Innovation Community

The concept of open innovation advocates for enterprises to actively cross organizational boundaries and seek external innovation resources, achieving complementarity between internal and external innovation resources. Since the 1970s, scholars, spearheaded by Professor Eric Von Hippel, have introduced a pioneering viewpoint that emphasizes the pivotal role of users as innovators in the innovation process [27]. This perspective has furnished enterprises with a theoretical foundation to incorporate user resources and engage in innovation. Prior studies on user contributions have concentrated on three primary themes. The first theme delves into the definition of user innovation contributions.

Initial scholars held the belief that user contribution behavior is demonstrated through active sharing of product or service usage experiences, with a focus on user initiative [3,28,29,30]. However, in recent years, a growing number of scholars have expanded the definition of user contribution to include the potential for user-driven creative implementation, implementation contribution, and product creative quality, among other factors. The second theme studies the reasons why users contribute to open innovation communities. Drawing on motivation theory and user satisfaction theory [31,32,33], the reasons may encompass individual intrinsic motivation, enthusiasm, psychological ownership of knowledge, and the desire for community recognition [34]. The third theme examines the features that influence users’ contributions to innovation. This research delves into the attributes of user ideas (quantity, popularity, length, and supporting evidence) [10,35,36], as well as the extent and variety of user interactions [8,34,37]. Through the lens of social networks, it examines the influence of network position and centrality on contributions [38,39,40], and the effects of collaborative innovation networks involving users, employees, businesses, and other stakeholders [41,42,43]. It is indisputable that user contributions hold significant importance for enterprises. While numerous studies have examined the potential influence of these factors on user contributions, there remains a dearth of research regarding the specific circumstances and mechanisms through which they exert an impact, Therefore, further investigation is warranted.

### 2.2. Personality Trait Theory

The personality trait theory holds that the trait is the fundamental characteristic that determines individual behavior. The “Big Five” of personality traits have been widely accepted by personality psychologists on the dimensional level. They include neuroticism, openness, extroversion, agreeableness, and conscientiousness. The five personality characteristics provide an entire general framework for understanding the influence of personality on behavior [44,45]. With the rapid advancement of the internet, the utilization of personality traits has extended beyond organizational contexts to non-organizational setting [46,47,48,49]. Scholars have begun to explore the application of individual personality traits in social media and virtual communities, research has demonstrated that personality traits influence individuals’ internet usage patterns, sense of community on websites, propensity to engage with social media platforms, willingness to participate in the metaverse, and other related behaviors [24,50,51].

### 2.3. User Engagement

Since the 21st century, the term “engagement” has been widely used in various disciplines, including sociology, political science, psychology, education, and organizational behavior. The concept of engagement originates from psychology, which refers to a state of conformity and adaptation. Scholars and enterprises have gradually realized that maintaining user engagement is crucial for retaining users and ultimately creating a competitive advantage for the enterprise. Prominent technology companies in the United States, such as Apple and Microsoft, have started to perceive ‘users’ and ‘user engagement’ as valuable resources, with the intention of converting them into potential sources of revenue in the future. This shift in focus signifies a strategic prioritization of aligning user preferences and needs with product offerings, with the aim of stimulating growth and profitability [52]. It can be seen that this concept is worth exploring in depth. The one-dimensional definition of user engagement pertains to a psychological state or behavioral expression [23,53], whereas the multidimensional definition offers a more comprehensive and widely accepted perspective, encompassing cognition, emotion, and behavior [54,55]. Within open innovation communities, users have the opportunity to share their opinions on products and services via this platform, and subsequently contribute innovative proposals to the community based on the issues encountered during the product experience. This interaction serves to strengthen the bond between users and the community [56]. Hence, this research posits that user engagement encompasses the cognitive, emotional, and proactive creative conduct exhibited by individuals within open innovation communities, which is shaped by their interactive encounters.

### 2.4. Impact of User Personality Traits on Innovation Contribution

Based on the “Big Five Personality” division in personality trait theory, each personality trait has different effects on innovation contribution. Conscientiousness is the strongest and most consistent predictor of cross-career and all success metrics. Highly conscientious people tend to be more motivated to perform well in their work [57,58]. Therefore, when users have conscientious personalities, they have higher achievement motivation in the open innovation community. They will make more efforts to achieve goals and will be more likely to implement the proposed ideas. Individuals with high openness tend to look for opportunities to learn new things and are more inclined to socialize through Facebook [59], Furthermore it is more likely to generate new product ideas [60,61], Therefore, the higher the score of users’ openness, the higher the possibility of ideas being implemented. Neurotic people are usually more emotionally unstable. Some scholars believe that people with high neuroticism scores have more negative comments on things and tend to reduce information-sharing behavior on social media [59]; However, some scholars have come to the opposite conclusion that neurotic users have more status updates in the online community, which can promote their information sharing and feedback exchange in the online community [62]. Therefore, the relationship between neuroticism and user contribution is still inconclusive. This study believes that the emotional instability of neuroticism users’ internal personalities, and their tendency to anger and anxiety, will lead users to post more emotionally, reduce the quality of ideas, and reduce the possibility of implementing the final idea. The measure of agreeableness is an individual’s prosocial nature, individuals who possess high levels of agreeableness tend to enhance their success in interpersonal relationships by utilizing Facebook as a means to connect with others [63]. Moreover, individuals with extroverted personalities, characterized by a strong inclination towards social attention, exhibit exceptional proficiency in managing situations that necessitate social interaction [64]. It is important to note that these two personality traits primarily pertain to interpersonal communication and do not directly influence user contributions in idea implementation. Consequently, based on the aforementioned analysis, Hypotheses 1–3 are formulated.

**Hypothesis** **1** **(H1).** *Conscientiousness positively predicts user contributions in idea implementation.*

**Hypothesis** **2** **(H2).** *Openness positively predicts user contributions in idea implementation.*

**Hypothesis** **3** **(H3).** *Neuroticism negatively predicts user contributions in idea implementation.*

### 2.5. The Mediating Role of User Engagement

Prior research has examined the mediating role of organizational factors, such as social capital, strategic orientation, and job satisfaction, in the association between personality traits and contribution. However, the potential mediating effects of user engagement have not been investigated. User engagement refers to the favorable cognitive, emotional, and behavioral responses that users exhibit towards a community when engaging with associated products and services. It has the capacity to introduce external knowledge, technology, and concepts to the organization, thereby facilitating product and service innovation. The incorporation of external knowledge, technology, and concepts into an enterprise can yield economic value, social value, and functional value during the process of product and service innovation. The significance of user engagement in the value co-creation process warrants consideration [65]. Consequently, diverse personality traits can engender varying levels of user engagement, subsequently influencing user innovation contributions. Hypotheses 4–7 are formulated.

**Hypothesis** **4** **(H4).** *Conscientiousness positively predicts user engagement.*

**Hypothesis** **5** **(H5).** *Openness positively predicts user engagement.*

**Hypothesis** **6** **(H6).** *Neuroticism negatively predicts user engagement.*

**Hypothesis** **7** **(H7).** *User engagement positively predicts user contributions in idea implementation.*

### 2.6. The Moderating Effect of Community Incentives

The trait activation theory seeks to explicate the manner in which suitable external contexts stimulate an individual’s inherent characteristics, thereby facilitating the manifestation of implicit traits in overt behavior [66]. Timothy (2015) posited that in occupations necessitating greater autonomy and ingenuity, the association between conscientiousness, openness, and job performance is more favorable [58]. The provision of community incentives can foster an environment conducive to innovation within the community [67]. In open innovation communities, the utilization of incentive mechanisms to encourage user participation in community activities is a crucial strategy for establishing and sustaining relationships with users, as well as enhancing user retention. The motivation within the community, as examined in this study, primarily pertains to fulfilling users’ elevated aspirations through spiritual motivation. Prior research has investigated the influence of community incentives on users’ willingness to participate and their contribution to value co-creation within virtual communities. Consequently, we posit that community incentives can effectively regulate the association between user personality traits and user engagement. Consequently, we put forward Hypotheses 8–10.

**Hypothesis** **8** **(H8).** *Community incentives positively predict the relationship between conscientiousness and user engagement.*

**Hypothesis** **9** **(H9).** *Community incentives positively predict the relationship between openness and user engagement.*

**Hypothesis** **10** **(H10).** *Community incentives positively predict the relationship between neuroticism and user engagement.*

**Figure 1:**
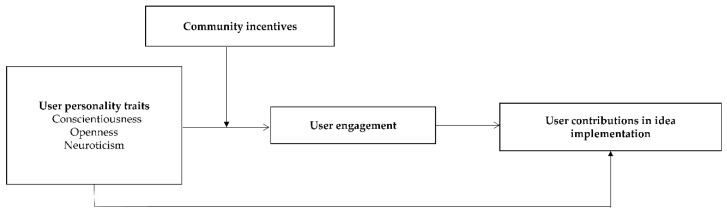
Proposed model of the study.

When users perceive support and motivation from the community, it can enhance the manifestation of their personality traits. Additionally, community incentives prompt users to establish stronger connections with the community, actively contribute feedback and creativity, and engage in behaviors that align with the community’s values [68]. Consequently, Hypotheses 11a-c are formulated. And our research model is shown in Figure 1.

**Hypothesis** **11a–c** **(H11a–c).** *Community incentives will moderate the strength of the mediated relationship between users’ conscientiousness (a), openness (b), and neuroticism (c) with their contributions in idea implementation via user engagement, such that the mediated relationship will be stronger under high community incentives than in under low community incentives.*

## 3. Research Methodology

### 3.1. Sample Selection and Data Source

This article selects the research object of Xiaomi’s open innovation community—MIUI Community (https://www.xiaomi.cn, accessed on 10 June 2022) The MIUI community is an open innovation community with a relatively successful operation, which has attracted a large number of “rice fans”. Xiaomi users can seamlessly connect to all product innovation and development aspects, such as idea solicitation, version testing, or marketing. Furthermore, the effective integration of users’ ideas and Xiaomi’s internal resources have promoted the iterative innovation of the MIUI mobile phone operating system. To sum up, the MIUI community can satisfy this article’s research on user contributions in idea implementation. As of 20 June 2022, we have collected users’ information in the “Mobile Phone” and “MIUI” sections of the MIUI community, and after deduplication of user IDs, we have selected 48,354 users who posted more than 10 ideas and whose ideas have been implemented as the research data for this paper.

### 3.2. Variable Measurement

#### 3.2.1. Personality Trait

Language can reflect people’s cognition, preference, and personality. Researchers can capture people’s characteristics by analyzing the types and frequency of words used in the language of experimental subjects [69]. Furthermore, digital information on social networks can predict users’ gender, age, personality, and other relevant information that has been widely verified [26,70]. How to measure users’ personality traits from Xiaomi community users’ posting texts is one of the studies focuses and innovations of this paper. The specific measurement methods are as follows:

Initially, the identification of word classes in psychology was conducted through the utilization of the Linguistic Inquiry and Word Count (LIWC) dictionary, as evidenced in Table 1. This dictionary, employed for quantitative analysis of textual words, encompasses 77 distinct word classes, comprising a total of 9049 words. Renowned for its commendable reliability and validity, the LIWC dictionary has garnered extensive utilization within the realm of psychology.

Subsequently, an investigation was undertaken to establish the correlation between LIWC feature parts of speech and the five personality traits. Andrew (2013) conducted a standard personality test to examine the correlation between each type of feature word in the LIWC dictionary and the big five personality traits [70]. We chose the correlation coefficient of significant correlation between LIWC characteristics and personality traits (*p* < 0.05) as the reference value of LIWC characteristics in each personality trait in the big five personality model, as shown in Table 2.

Finally, the score of personality traits was calculated by taking the following steps:

- Text cleaning. Firstly, we perform text cleaning on the user’s original text content, removing numerical numbers, repeated but meaningless keywords, etc.
- Text segmentation. The “Jieba” tool, a robust Chinese word segmentation tool, is employed to segment user-posted information into individual words, convert it into pure phrase text, and calculate the overall word frequency [71]. The length of the segmented phrase is recorded as n.
- Phrase matching. If the word segmentation result is retrieved and makes a hit in the LIWC dictionary, the corresponding label will be directly assigned to the phrase; otherwise, semantic similarity will be considered for classification. We considered using the open-source pre-training word vector model “w2v. baidu_encyclopedia. target. word. dim300” provided by Baidu Paddle NLP to calculate the word vectors of all words under various feature word tags. And based on the above pre-training word vector model, the word vector of the phrase that does not make a hit in the LIWC dictionary retrieval is obtained. And then, the cosine similarity between the spatial position of these feature words and some existing feature tags is calculated, and the feature classification tag with the highest cosine similarity is chosen as the final feature classification tag.
- Count word frequency. Based on the result of phrase matching, LIWC is used to count the word frequency of each type of feature word proposed by users.
- Score calculation. According to the assignment result of the tag of characteristic words and the mapping relationship between the LIWC characteristic words in the table and the five personality characteristics, the user’s score in each type of personality trait dimension is calculated and recorded as P_i_ (i = {O, C, E, A, N}). The overall formula is $P_{i}=\frac{\sum_{j=1}^{77}K_{ij}*F_{j}}{n}$ i = {O, C, E, A, N}, j ∈ {1,77}. Among them, K_ij_ is the correlation coefficient between the j-type characteristic words and the i-type personality, and Fj is the word frequency of the j-type characteristic words.

#### 3.2.2. User Contributions in Idea Implementation

The dependent variable in this study pertains to the extent of the implementation contribution made by users to the enterprise. Within the MIUI new product development community, Xiaomi’s internal experts and members of the MIUI development team inform users about the progress of their ideas by placing a stamp in the upper right section of the thread. We have verified that the four stamps, namely optimized, under development, approved, and resolved, signify the adoption and implementation of these ideas by the company. The number of proposals published by users that were successfully implemented by enterprises serves as an indicator of their creative contribution [8].

#### 3.2.3. User Engagement

User engagement refers to the extent of alignment between users and the community, encompassing cognitive, emotional, and behavioral dimensions. This alignment is assessed through the examination of user interaction traces within the community. Cognitive engagement is gauged by the average word count of users’ posts, while emotional alignment is determined by the total number of posts expressing positive emotions. Additionally, behavioral alignment is evaluated by considering the overall number of posts contributed by users within the community. To quantify user engagement, the mean of these three dimensions is employed [56].

#### 3.2.4. Community Incentives

In the Xiaomi community, the community will support and motivate users by publishing medals. In this article, community incentives are measured by the number of medals received by users [72].

#### 3.2.5. Control Variable

The control variable chosen for this study was community tenure, defined as the duration in months between the user’s initial post in the community and the data collection period [11].

### 3.3. Data Analysis

#### Descriptive Statistics, Correlation Analysis, and Collinearity Analysis

First, descriptive and correlation analyses were conducted to visually understand the sample data. Table 3 shows the descriptive statistical results, with a total sample size of 48,354. Table 3 also shows the basic correlation among the variables. In order to ensure the validity of the model, we tested VIF values of each variable and found that they are all less than 5, indicating that there is no issue of multicollinearity among the variables.

## 4. Data Analysis and Results

### 4.1. Impact of Personality Traits on User Contributions in Idea Implementation

We used Stata 17 for data analysis and hypothesis testing. Given the countable nature of the dependent variable and the discrete nature of the data, negative binomial regression was employed for model testing. M1 was used to assess the influence of personality traits on the contributions in idea implementation and the results are shown in Table 4. The findings indicated that higher levels of conscientiousness in user personality were associated with greater contributions in idea implementation (β = 0.123, *p* < 0.001); the extent of openness in an individual’s personality positively correlates with the level of contribution they make to idea implementation (β = 0.101, *p* < 0.001). Conversely, a higher level of neuroticism in an individual’s personality negatively affects their contribution to creative implementation (β = −0.052, *p* < 0.001), H1–H3 are supported. The inherent traits of openness and conscientiousness in individuals stimulate their internal motivation to generate high-quality ideas and enhance their overall contribution. However, neurotic personality traits, characterized by emotional instability, do not contribute to creative implementation, and may even hinder it.

### 4.2. Mediation of User Engagement

The above study M1 has confirmed a significant relationship between three personality traits and user contributions in idea implementation, Additionally, study M2 has corroborated the association between these three personality traits and user engagement. Furthermore, study M3 has verified the influence of these three personality traits on user implementation contribution, taking into account the mediating effect. As depicted in Table 4, conscientious personality (β = 0.176, *p* < 0.001), open personality (β = 3.564, *p* < 0.001), and neurotic personality (β= −6.585, *p* < 0.001) exhibit a substantial influence on user engagement, thereby providing support for Hypotheses 4–6. Upon considering the inclusion of the mediating effect, a significant relationship is observed between conscientious personality (β = 0.084, *p* < 0.001), open personality (β = 0.026, *p* < 0.01), neurotic personality (β = 0.033, *p* < 0.01), and user contributions in idea implementation. The mediating variable, user engagement, exerts a favorable influence on user implementation contribution. The validation of Hypothesis 7 signifies that user engagement assumes a partial mediating function in the connection between conscientious personality, open personality, neurotic personality, and user implementation contribution. User engagement is a dynamic and interactive connection that is established between users, enterprises, and other users during the process of interaction and value co-creation. The formation of user engagement can be influenced by the personality traits of users, and in turn, user engagement can enhance the contribution of users in the implementation process. User engagement serves as a partial intermediary in this relationship.

### 4.3. Moderator of Community Incentives

The impact of interaction items involving conscientiousness, openness, neuroticism, and community incentives on user engagement was examined by M4, M5, and M6, respectively. The findings of the moderating effect are presented in Table 4, indicating that community incentives positively moderate the association between conscientious personality and user engagement (β = 1.666, *p <* 0.001). Conversely, negative moderation is observed in the relationships between open personality (β = −0.412, *p <* 0.001), neurotic personality (β = −3.424, *p <* 0.001), and user engagement. The conscientious personality exhibits a propensity for adhering to rules, striving for achievement, and valuing community motivation. This inclination serves as a catalyst for fostering a strong connection with the community, consequently positively influencing its relationship with users. Conversely, the open personality tends to gravitate towards independent and innovative work, potentially perceiving the incentive mechanism of the community as a hindrance to their focus on innovative activities. Consequently, this dynamic negatively impacts their user engagement. Additionally, individuals with a neurotic personality are susceptible to feelings of anxiety and anger, rendering them ill-equipped to adapt to the environment fostered by the community. Consequently, Hypothesis 8 was confirmed, while Hypotheses 9 and 10 were not supported.

### 4.4. The Moderated Mediation Effect

We used Model 7 of the Process program in the SPSS.27 software for testing and used the Bootstrap method with a sample size of 5000 to verify the moderated mediating effect while controlling for user community tenure, as shown in Table 5 and Table 6. The coefficient of the interaction term between conscientious personality and community incentives in Table 5 is significantly positive. Conversely, the interaction term between openness, neuroticism, and community incentives is significantly negative. Table 6 presents empirical evidence indicating that the augmentation of community incentives leads to a heightened influence of user engagement on the association between conscientious personality and implementation contribution. This finding suggests that community incentives play a constructive role in moderating this relationship. Additionally, the 95% confidence interval [0.004–0.011] excludes the null value of 0, thereby establishing the significance of the moderated mediating effect and providing support for Hypothesis 11a. Furthermore, the mediating effect of community incentives in moderating user engagement between open personality and implementation contribution falls within the 95% confidence interval [−0.008–0.004], which includes 0, indicating that the moderated mediation effect is not significant, and hypothesis 11b is not supported. The results of the analysis suggest that community incentives have a significant moderating effect on the relationship between neurotic personality and implementation contribution. The 95% confidence interval for this effect, [−0.028–0.011], does not include 0, indicating its statistical significance. However, it is important to note that the coefficient is negative, indicating that as community incentives increase, the mediating effect of user engagement decreases, indicating a negative moderating effect. Therefore, Hypothesis 11c is not supported.

## 5. Discussion

### 5.1. Theoretical Contribution

This study made significant advancements in understanding the individual factors that influence user contributions in idea implementation. It introduced personality traits from the field of psychology into the realm of user innovation, thereby enhancing the existing body of research on user innovation behavior. Currently, scholarly investigations predominantly focus on exploring the correlation between individual personality traits and various life and work behaviors [48,73], such as perceived happiness, career success, job burnout, as well as community participation and information sharing behaviors within virtual communities [74,75]. This study investigated the influence of individual personality traits on the extent to which users contribute to idea implementation within open innovation communities, thereby extending the application of personality trait theory. Additionally, this study presents unique findings that diverge from those observed in other organizational contexts. Specifically, within open innovation communities, conscientiousness and openness exhibit a substantial positive effect on contributions in idea implementation. Notably, the examination of neuroticism reveals a negative impact on creative implementation, as evidenced by direct testing. The positive impact of neuroticism on implementation contributions was found to be mediated by user engagement, thereby enhancing the discourse on neurotic personality. This research outcome introduces a boundary mechanism that elucidates the positive influence of neuroticism on performance. Specifically, the presence of a positive impact on implementation contribution is contingent upon the mediating effect of user engagement, indirectly supporting the “loneliness theory” which posits that individuals with high neuroticism tend to utilize the internet as a means to alleviate loneliness. The findings of this study indicate that neurotic personality traits can enhance user innovation contribution through the mediating influence of user engagement [76,77,78]. This offers a novel theoretical framework for comprehending the behavior of neurotic users.

Additionally, this research advances our understanding of the mediating and moderating mechanisms linking individual personality traits to idea implementation contributions and enhances our knowledge of the impact pathway through moderated mediating mechanisms. Furthermore, this study extends the concept of customer engagement in marketing to open innovation communities, presenting new insights. The findings of the study suggest that user engagement plays a partial mediating role in the association between personality traits and idea implementation contributions, thereby adding to the existing body of research. Additionally, the investigation of the moderating effect of community incentives revealed that these incentives have a positive regulatory effect on the link between conscientious personality and user engagement, a negative regulatory effect on the association between openness and neurotic personality with user engagement, and a positive regulatory effect on the influence of user engagement on the relationship between conscientious personality and idea implementation contributions. It has been confirmed that the community incentive environment has the capacity to stimulate the latent driving force of conscientiousness, foster the development of user engagement, augment the contribution of user idea implementation, and enrich the empirical investigation of trait activation theory. The examination of this impact pathway offers theoretical direction on how to enhance the contribution of user idea implementation.

Moreover, in terms of research methodology, the measurement of personality traits was objectively conducted by analyzing textual information derived from user posts and comments within the community. The personality traits scores were derived using text mining and machine learning techniques, which replaced the conventional approach of questionnaire surveys for measurement. The objectivity of the personality traits assessment obtained through this methodology is noteworthy. Text mining and machine learning have emerged as potent tools for predicting human behavior and personality traits, finding extensive application in the analysis of social network data, including company annual reports and Weibo [79]. The present study specifically concentrates on user text messages within the Xiaomi community to identify and characterize user personality traits, thereby introducing a novel paradigm and methodology for data collection in psychological research on personality trait recognition.

### 5.2. Practical Enlightenment

This research is based on the user group of the Xiaomi Community, which has a certain reference significance for the open innovation community built by enterprises. First, the research shows that openness, conscientiousness personalities are significantly and positively related to innovation contributions. As a result, community managers can identify user personality traits through user messages in the community and attract users with high openness, conscientiousness, to become leading users and pay more attention to their ideas. By opening the inner measuring circle, these users will be more exposed to the latest products of the enterprise and put forward more ideas. Secondly, different measures should be taken for users with different personalities in community management. For individuals with high levels of conscientiousness, incentive measures such as points and rewards can be utilized to provide them with premium motivational benefits, foster increased interaction and engagement within the community, and amplify the contributions made by users with conscientious personalities. Regarding open personalities, community motivation is not a motivating factor. Instead, creating a more independent atmosphere can help users feel more comfortable and fit in. Conversely, allowing them to participate in too many community activities may have the opposite effect. For individuals with neurotic personalities, enhancing their creative implementation contribution requires creating user engagement and increasing investment in the community at cognitive, emotional, and behavioral levels. Ultimately, for users to optimize their experience in a community, it is crucial for them to comprehend their own personality traits and engage selectively in community activities. This approach can facilitate the enhancement of their creative implementation contributions.

### 5.3. Limitations and Future Direction

This study explores the impact mechanism of users’ personality traits on users’ contributions in idea implementation. However, there are still some limitations. First, text mining and machine learning methods were innovatively used to measure personality traits, and their accuracy needs further verification. In subsequent studies, comparative verification can be conducted by issuing questionnaires. Secondly, this study selects the sample of the Xiaomi community for research. In the future, other open innovation communities can be investigated to further increase the reliability of the study. Furthermore, research indicates that user engagement is part of the mediation between openness, conscientiousness, neuroticism personality and user contribution, and other mediation have not been confirmed. In order to effectively manage users with varying personality traits and enhance overall innovation contribution within communities, it is imperative to comprehend the distinct impact pathways through which these traits influence innovation contribution. Thus, future research endeavors should focus on investigating additional mediating mechanisms, including the influence of user cognition, emotions, and other relevant factors on behavior. In addition, the trait activation theory proposes three levels of situational functions: task, social, and organizational. This study exclusively examined the effects of community incentives within organizations on the correlation between personality traits and behavior. Future research should delve deeper into the role of situational factors, including task-level product iteration speed and social-level community interpersonal interactions, in shaping the relationship between personality and behavior.

## 6. Conclusions

This study investigates the influence of user personality traits on their involvement in idea implementation within open innovation communities. The empirical analysis demonstrates that individuals with elevated levels of conscientiousness and openness exhibit greater contributions to idea implementation, whereas those with higher levels of neuroticism tend to contribute less. Furthermore, user engagement is found to partially mediate the association between personality traits and the extent of idea implementation contribution. Importantly, this study presents significant findings concerning neurotic users, particularly under the mediating influence of user engagement, neurotic personality is positively associated with idea implementation contributions, this highlights the significance of user engagement in facilitating this relationship. Additionally, the study emphasized the role of community incentives, based on trait activation theory. The results indicated that community incentives positively moderate the relationship between conscientious personality and user engagement, as well as the mediating effect of user engagement on the relationship between conscientious personality and idea implementation contributions.

These findings illustrate a distinct pathway linking personality traits to contributions in innovation, thereby extending the theoretical application of personality trait frameworks. For the first time, this research substantiates the magnitude and pathway of influence that diverse personality traits exert on user idea implementation contributions, enriching and refining research in the field of user innovation. The investigation into community incentives further augments the application of trait activation theory. Concurrently, the validation of the moderated mediation model posited within this study offers insights into the manner in which personality traits impact innovative contribution. Consequently, it offers valuable insights for open innovation community managers, enabling them to comprehend the intricate interplay between user personality and their potential contributions. Ultimately, this understanding can facilitate more effective user-community interactions and guide managers in implementing personalized strategies, thereby fostering the enhancement of user implementation contributions.

## Figures and Tables

**Table 1:** Examples of Chinese version of LIWC2015 dictionary (part).

Characteristic Word Category	Example	Number of Words
Pronoun	I, them, myself	104
Negative word	No	111
Positive emotion words	Joy, love, merit	730
Anxious words	Anxiety, fear	169
Angry words	Anger, revenge, hate	358
……	……	……
Leisure words	Travel, vacation, hobbies	462

**Table 2:** Correlation coefficient between LIWC feature words and big five personality traits (part).

LIWC Category	E	A	C	N	O
Pronoun	0	0	−0.03 ***	0.04 ***	0.07 ***
Negative word	−0.06 ***	−0.05 ***	−0.03 ***	0.07 ***	0.02 ***
Positive words	0.13 ***	0.13 ***	0.1 ***	−0.08 ***	−0.07 ***
Anxious words	−0.04 ***	−0.02 ***	−0.12 ***	0.06 ***	0.07 ***
Angry words	−0.05 ***	−0.19 ***	−0.12 ***	0.11 ***	0.02 ***
……	……	……	……	……	……
Leisure words	0.06 ***	0.04 ***	0.03 ***	−0.07 ***	0

*** At the 0.001 level, the correlation is significant.

**Table 3:** Descriptive statistics, correlation analysis, and Collinearity analysis.

Variable	Mean	SD	1	2	3	4	5	6	7	VIF
Contribution	6.213	7.027	1							1.152
Engagement	39.905	38.287	0.309 ***	1						1.234
C	2.650	0.948	0.074 ***	0.071 ***	1					2.922
O	3.311	0.708	−0.022 ***	−0.019 ***	−0.652 ***	1				2.268
N	1.210	0.661	−0.057 **	−0.083 ***	−0.805 **	−0.739 ***	1			3.707
incentives	21.135	13.635	0.251 ***	0.358 ***	0.137 ***	−0.080 ***	−0.146 ***	1		1.573
tenure	28.365	25.464	0.054 ***	0.246 ***	0.075 ***	0.008	−0.060 ***	0.052 ***	1	1.415

*** At the 0.001 level, the correlation is significant. ** At the 0.01 level, the correlation is significant.

**Table 4:** Hierarchical regression results.

	M1	M2	M3	M4	M5	M6
Conscientiousness	0.123 ***	0.176 ***	0.084 ***	1.233 ***		
Openness	0.101 ***	3.564 ***	0.026 **		0.188 ***	
Neuroticism	−0.052 ***	-6.585 ***	0.033 **			−2.859 ***
User engagement			0.018 ***			
C* incentives				1.666 ***		
O* incentives					−0.412 ***	
N* incentives						−3.424 ***
Community incentives				0.843 ***	0.880 ***	0.783 ***
Community tenure	0.002 ***	0.359 ***	−0.002 ***	0.126 ***	0.123 ***	0.136 ***
Constant term	1.162 ***	25.432 ***	0.694 ***	15.012 ***	17.160 ***	22.441 ***
R^2^	0.003	0.672	0.060	0.134	0.133	0.141

*** At the 0.001 level, the correlation is significant. ** At the 0.01 level, the correlation is significant. C* incentives represent the interaction term between Conscientiousness and Community incentives, O* incentives represent the interaction term between Openness and Community incentives, N* incentives represent the interaction term between Neuroticism and Community incentives.

**Table 5:** Moderated mediation effect test.

	Conscientiousness			Openness			Neuroticism		
	β	SE	t	β	SE	t	β	SE	t
Personality	−0.1492	0.307	−4.866 ***	1.090	0.408	2.669 **	5.173	0.429	12.066
incentives	0.501	0.042	11.842 ***	1.021	0.060	17.115 **	1.243	0.023	53.540 ***
Per *incentives	0.129	0.014	9.397 ***	−0.043	0.018	−2.319 *	−0.380	0.019	−20.144 ***
Com tenure	0.126	0.007	16.853 ***	0.123	0.008	16.343 ***	0.136	0.007	18.253 ***
Constant term	22.234	0.880	25.261 ***	14.175	1.374	10.318 ***	12.720	0.588	21.638 ***
R^2^	0.135			0.133			0.141		

*** At the 0.001 level, the correlation is significant. ** At the 0.01 level, the correlation is significant. * At the 0.05 level, the correlation is significant.

**Table 6:** Indirect effect test results under different community incentive levels.

		Effect	SE	LLCI	ULCI
Conscientiousness	Low level	−0.030	0.018	−0.069	0.002
	Average value	0.071	0.013	0.043	0.092
	High level	0.171	0.035	0.104	0.243
	Moderated Mediation index	0.007	0.002	0.004	0.011
Openness	Low level	0.044	0.028	−0.018	0.086
	Average value	0.011	0.019	−0.024	0.047
	High level	−0.023	0.060	−0.115	0.094
	Moderated Mediation index	−0.002	0.003	−0.008	0.004
Neuroticism	Low level	0.133	0.044	0.046	0.201
	Average value	−0.164	0.026	−0.205	−0.105
	High level	−0.461	0.085	−0.582	−0.263
	Moderated Mediation index	−0.022	0.005	−0.028	−0.011

## Data Availability

The data presented in this study are available on request from the corresponding author.
